# Association between health-related physical fitness and incident hypertension among the elderly in Wuhan: a seven-year cohort study

**DOI:** 10.1186/s12889-025-25635-3

**Published:** 2025-11-27

**Authors:** Wen Luo, Yuqian Zhang, Xinghua Liu, Yaqiong Yan, Wei Zhang, Sisi Ke, Siqi Zhao, Tonghui Yuan, Liyin Wang, Mei Yang, Yan Guo

**Affiliations:** 1 Wuhan Centre for Disease Control and Prevention, Wuhan, China; 2 School of Public Health, Wuhan University of Science and Technology, Wuhan, China; 3 Department of General Surgery, Union Hospital, Tongji Medical College, Huazhong University of Science and Technology, Wuhan, China

**Keywords:** Hypertension, Health related physical fitness, Cohort study, Elder population

## Abstract

**Aim:**

This study aimed to establish robust epidemiological evidence on the association between various dimensions of health-related physical fitness (HRPF) and hypertension among older adults.

**Methods:**

We conducted a large-scale, 7-year cohort study. The baseline survey was conducted in 2015 among participants aged 65 or above from Wuhan, China. Follow-up assessments were integrated into an annual physical examination program for elderly residents in Wuhan, with outcome data collected in 2018, 2019, 2020, 2021, and 2022, respectively. The association between HRPF and hypertension was evaluated using chi-square tests and multivariate Cox regression analyses.

**Results:**

Hypertension status was tracked in 695 participants, with an incidence rate of 36.4%. Individuals who developed hypertension showed significantly poorer performance in the chair sit-and-reach (2.60 ± 0.89 VS 2.8 ± 1.13, *P* = 0.028) and the 30 s arm curl (2.13 ± 1.16 VS 2.26 ± 0.93, *P* = 0.024), but better performance in back scratch (3.53 ± 1.05 VS 3.74, *P* = 0.009). Multivariate Cox regression analysis revealed that better performance in the 30-second chair stand test (β:-0.155, HR:0.86, 95%CI: 0.74–0.99, *P* < 0.05) and back scratch test (β:-0.140, HR:0.87, 95%CI: 0.77–0.97, *P* < 0.05) was associated with a reduced risk of hypertension, indicating that both are protective factors against the development of hypertension.

**Conclusions:**

Better lower-body muscle strength and upper-body body flexibility appear to be associated with a lower risk of hypertension. Exercise programs targeting these dimensions are recommended to reduce the incidence of hypertension among older adult.

## Introduction

Hypertension represents a serious public health issue warranting high priority in prevention strategies [1]. In China, the prevalence of hypertension among individuals aged 60 and older reached 52.6% in 2022 [2]. For the elderly people over 65, hypertension is the leading risk factor for cardiovascular disease (CVD), stroke and all-cause mortality. Hypertensive heart disease has risen in rank from 17th to 10th among the causes of years of life lost (YLL) [3]. Hypertension also poses a significant economic burden on families and society. The average direct medical costs and cost of illness were $ 467.2 and $ 9393.3 in rural areas, and the total cost of hypertension in one remote province was around $ 231.7 million [4, 5]. Therefore, identifying effective preventive measures for hypertension in the elderly is of urgent importance.

It is well established that that regular physical activity has benefits on blood pressure control [2]. However, the effects of different types of physical exercise on blood pressure control vary. For instance, aquatic exercise and moderate-intensity activities appear to be more effective in reducing blood pressure than land-based or high-intensity exercises [6–8]. Similarly, isometric handgrip and leg exercise have greater effect sizes in reducing diastolic blood pressure and systolic blood pressure compared to endurance training and dynamic resistance training [9]. Preliminary studies have shown that hypertensive patients exhibit performance differences in health-related physical fitness(HRPF), such as one-leg balance, two-minute step test, and back scratch test—suggesting that distinct exercise modalities may influence blood pressure through improvements in specific dimensions of HRPF [10, 11]. 

HRPF is defined as the ability to remain energetic and alert during daily activities without feeling excessively fatigued, while also being capable of enjoying leisure time interests and coping with unpredictable emergencies [12, 13]. As an outcome of physical activity, HRPF can also serve as a reference for exercise guidance. It includes five aspects, body composition, muscular strength, muscular endurance, flexibility, and cardio-respiratory fitness [12–14]. Existing evidence suggests that better body flexibility and higher muscle strength are associated with a reduced risk of hypertension [15]. – [16] Furthermore, cardio-respiratory fitness shows an inverse association with the occurrence of hypertension by using a non-exercise algorithm [17]. However, most existing studies have not comprehensively assessed HRPF, often focusing on only one or two components of HRPF, with insufficient research evaluating flexibility [14]. To address this gap, we propose the hypothesis that different dimensions of HRPF exhibit distinct relationships with hypertension and conducted a large-scale 7-year cohort study to examine these associations.

## Materials and methods

### Study design and participants

This cohort study was conducted by the Department of Disease Burden Innovation Research at the Wuhan Centre for Disease Control and Prevention.

The baseline survey was conducted in 2015. Participants were selected from 3 to 5 communities within 7 of Wuhan’s 17 administrative districts using multi-stage stratified random sampling method. Inclusion criteria consisted of permanent community residents aged 65 years or older who signed the informed consent form. Exclusion criteria included individuals with severe illnesses that precluded participation in HRPF assessments, as well as those diagnosed with severe mental illnesses.

Follow-up was integrated into the annual physical examination survey for older adults in Wuhan, with outcome data collected in 2018, 2019, 2020, 2021, and 2022, respectively. Given a substantial loss to follow-up (26.8%) due to relocation, loss of contact, death, and inability to participate in HRPF measurements, we evaluated potential selection bias by comparing baseline characteristics between retained and lost-to-follow-up participants. The results indicated no significant differences in general characteristics between the two groups.

### General characteristics assessment

Baseline characteristics were collected using a structured questionnaire and physical examination conducted by trained professionals. The questionnaire covered sociodemographic information (e.g., sex, age, marital status), lifestyle factors (e.g., physical activity, smoking status), and medical history (including physician-diagnosed hypertension, diabetes mellitus, stroke, cardiovascular disease, hyperlipidemia, and fall injuries). All investigators received standardized training and administered questionnaires following uniform protocols. The physical examination included basic anthropometric measurements such as height, weight, waist circumference, and blood pressure.

### Health Related-Physical fitness (HRPF) assessment

HRPF was assessed according to the American College of Sports Medicine (ACSM)’s Guidelines for Exercise Testing and Prescription (Ninth Edition) [18]. The evaluation included seven indicators across four dimensions.

Aerobic endurance was measured using the 2-minute step test, during which participants stepped in place, raising their knees to mid-patella height.

Muscle strength was assessed through the 30-second arm curl test and the 30-second chair stand test. In the former, participants performed seated elbow flexion with dumbbells; in the latter, they rose to a full stand from a seated position with arms crossed over the chest.

Flexibility was evaluated using the chair sit-and-reach test and the back scratch test. For the chair sit-and-reach test, participants sat with legs extended and reached forward with their fingertips; the distance from fingertips to toes was recorded. The back scratch test measured the spinal rotation and overlapping reach distance between hands behind the back.

Balance was assessed via the 8-foot up-and-go test and the one-leg balance test. The 8-foot up-and-go test required participants to stand up, walk a distance of 8 feet (approximately 2.44 m), and return as quickly as possible. The one-leg balance test measured the duration a participant could maintain balance while standing on the non-dominant foot with eyes open.

HRPF assessments were administered by certified healthcare professionals or trained community health workers. Participants were instructed to perform each test without excessive exertion; inability to complete a test independently was documented. Prior to formal assessment, practice trials were conducted for each test to ensure standardization of the measurement procedures. Equipment used included: timing devices, distance measuring instruments (with a precision of 0.1 cm), chairs with backrests (seat height: 43 cm), dumbbells (3.6 kg for men, 2.25 kg for women), and marker cones.

### Outcomes assessment

The primary endpoint was defined as either: (1) new-onset hypertension, or (2) the end of follow-up whichever occurred first. New-onset hypertension was identified based on any of the following: use of anti-hypertensive medication, self-reported physician diagnosis, or blood pressure values exceeding 140/90 mmHg during routine physical examinations. Follow-up termination occurred in cases of non-hypertensive death or loss to follow-up. Mortality data were obtained from the Wuhan Death Surveillance System. Loss to follow-up was operationally defined as failure to contact the participant after at least three attempts to contact them via home visits, telephone calls, or inquiries through community health workers. The follow-up duration was calculated from the baseline survey until either the diagnosis of hypertension or the end of follow-up.

### Statistical analysis

Statistical analyses were performed using R software (version 4.3.1). Continuous data are presented as mean ± standard deviation, and group differences were assessed using chi-square tests. Multivariate cox regression analysis was conducted to evaluate the association between each physical fitness indicator and the incidence of hypertension. To systematically control for potential confounders, four sequential models were constructed based on prior clinical knowledge and established epidemiological principles: Model 1 was unadjusted; Model 2 was adjusted for sociodemographic factors (gender and age); and Model 3 was further adjusted for health-related covariates (exercise, smoking, and fall history); Model 4 was adjusted for Height, Weight, BMI, Hip Circumference, WHR and Heart Rate. The proportional hazards assumption for all Cox models was verified using schoenfeld residuals tests, and no substantial violations were observed. Statistical significance was defined as a two-sided P-value < 0.05.

### Covariates assessment

Covariates included participants’ general characteristics collected via a structured questionnaire, covering name, age, gender, marital status, educational background, history of chronic illnesses (including hypertension, diabetes, stroke, cardiovascular disease, hyperglycemia, fall, and other primary chronic conditions), physical activity levels, and smoking habits. Physical measurements— height, weight, body mass index, waist circumference, hip circumference, waist-to-hip ratio (WHR), heart rate, and blood pressure—were obtained by qualified nurses during physical examinations.

Additional covariates relevant to HRPF and hypertension included sociodemographic characteristics and health-related factors. For this study, age, gender, physical exercise, smoking status, and history of falls were incorporated into the adjusted models.

## Result

### General characteristics of participation

**Fig. 1:**
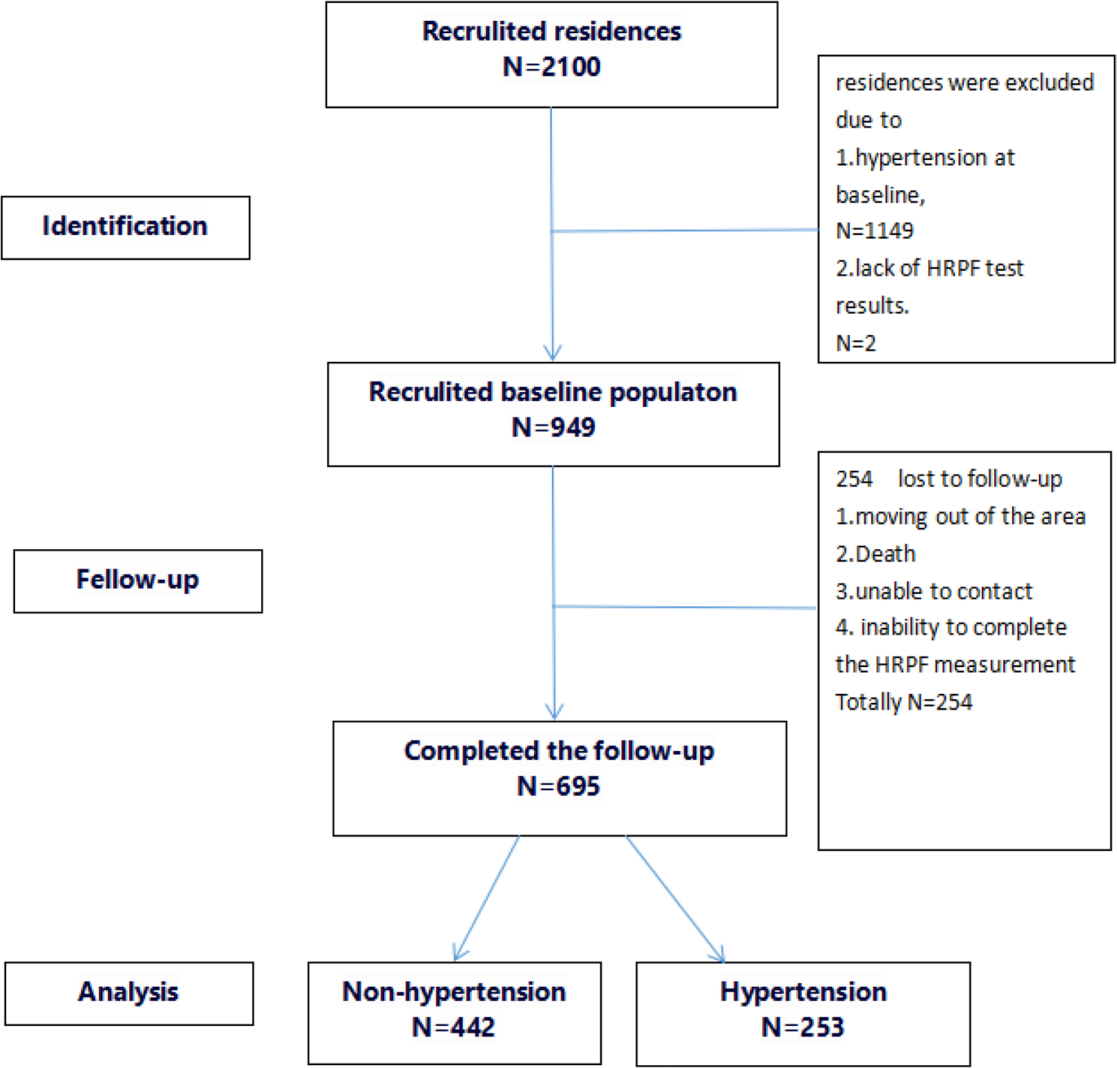
Flow chart of cohort study

The baseline survey initially included 2100 residences. Among them, 949 were enrolled for follow-up in this study. A total of 1149 residences were excluded due to pre-existing hypertension at baseline, and 2 additional participants were excluded due to lack of HRPF test results. Of the 949 eligible participants, 254 were lost to follow-up during the study period. Reasons for loss to follow-up included relocation outside the study area, non-hypertension-related deaths, and unable to contact. Ultimately, 695 participants completed the follow-up and were included in the final analysis. (Fig. 1)

The general characteristics of participation are presented in Table 1. The average age of sample was 72.4 years. A total of 267 participants (28.1%) reported lack of physical exercise, 190 (20.0%) reported being regular smokers, and 98(10.3%) reported a history of fall-related injuries.

Compared with males, females exhibited a significantly lower prevalence of smoking, as well as lower height, weight, body mass index (BMI), systolic blood pressure (SBP), and diastolic blood pressure (DBP). Conversely, females showed a higher incidence of falls.

**Table 1:** General characteristics of participation

	**N=949**	**Male **	**Female **	*p*
		(n=479, 50.47%)	(n=470, 49.53%)	
Age (years)	72.40 (5.18)	72.60 (5.16)	72.10 (5.20)	0.091
Lack of exercise (n, %)				
Yes	267(28.10%)	126(26.30%)	141(30.00%)	0.233
No	682(71.90%)	353(73.7%)	329(70.00%)	
Smoking (n, %)				
Yes	190 (20.00%)	181 (37.80%)	9 (1.90%)	＜0.001
No	759 (80.00%)	298 (62.20%)	461 (98.10%)	
Fall (n, %)				
Yes	98 (10.30%)	30 (6.30%)	68 (14.50%)	＜0.001
No	849 (89.50%)	448 (93.50%)	401 (85.30%)	
Height (m)	1.60 (0.08)	1.65 (0.06)	1.54 (0.06)	＜0.001
Weight (Kg)	60.50(10.70)	64.10 (10.40)	56.80 (9.63)	＜0.001
BMI (kg/m^2^)	23.60 (3.40)	23.40 (3.37)	23.70 (3.43)	0.049
WC (cm)	86.10 (9.59)	86.50 (9.32)	85.70 (9.84)	0.086
HC (cm)	95.20 (8.46)	95.20 (8.23)	95.10 (8.70)	0.765
WHR	0.91 (0.06)	0.91 (0.06)	0.90 (0.06)	0.051
Heart Rate(beats/min)	76.60 (10.80)	76.90 (11.40)	76.20 (10.2)	0.29
BP (mmHg)				
SBP	135.00 (18.50)	137.00 (19.20)	133.00 (17.60)	0.047
DBP	79.10 (10.70)	80.40 (11.10)	77.70 (10.10)	＜0.001

*WC* Waist Circumference, *HC* Hip Circumference, *WHR* Waist-to-Hip Ratio, *BP *Blood Pressure *SBP* Systolic Blood Pressure, *DBP* Diastolic Blood Pressure

### Health-Related physical Fitness(x ± SD)

The HRPF profiles of the participants are summarized in Table 2. Compared with male, females showed significantly poorer performance in one-leg balance test (1.36 VS 1.56, *P* = 0.009), 30 s chair stand (2.57 VS 2.81, *P* < 0.001), chair sit-and-reach (4.64 VS 4.52, *P* = 0.008), 30 s arm curl (2.11 VS 2.25, *P* = 0.015), 2 min step (2.44 VS 2.64, *P* = 0.011).

**Table 2:** Health-Related Physical Fitness Profiles of the Participants

	**N=949**	**Male **	**Female**	*p*
		(n=479, 50.47%)	(n=470, 49.53%)	
One-Leg Balance (s)	1.46 (0.97)	1.56 (1.11)	1.36 (0.79)	0.009
30 Seconds Chair Stand (reps)	2.69 (1.07)	2.81 (1.10)	2.57 (1.03)	<0.001
Chair Sit-and-Reach (cm)	4.58 (1.03)	4.52 (1.02)	4.64 (1.03)	0.008
30 Seconds Arm Curl (reps)	2.18 (0.99)	2.25 (1.06)	2.11 (0.90)	0.015
2 Minutes Step (reps)	2.54 (1.04)	2.64 (1.07)	2.44 (1.00)	0.011
Back Scratch (cm)	3.59 (1.07)	3.55 (1.05)	3.63 (1.10)	0.2
8-Foot Up-and-Go (s)	2.82 (1.03)	2.81 (1.04)	2.83 (1.02)	0.386

### Association between Health-Related physical fitness and incident hypertension

Hypertension status was successfully tracked in 695 participants during the follow-up period.(Table 3) Among these, 253 individuals developed hypertension, the incidence of hypertension is 36.4%. Compared to those without hypertension, participants who developed hypertension demonstrated significantly poorer performance in chair sit-and-reach (2.60 ± 0.89 VS 2.80 ± 1.13, *P* = 0.028) and 30 s arm curl (2.13 ± 1.16 VS 2.26 ± 0.93, *P* = 0.024). Conversely, they exhibited better performance in back scratch (3.53 ± 1.05 VS 3.74, *P* = 0.009).

**Table 3:** Participants HRPF and Hypertension Incident

	N=695	**hypertension**	**Non-hypertension **	*p*
		** (n=253, 36.4%)**	(n=442, 63.6%)	
One-Leg Balance (s)	1.48 (0.98)	1.53 (1.03)	1.46 (0.95)	0.426
30 Seconds Chair Stand (reps)	2.73 (1.05)	2.60 (0.89)	2.80 (1.13)	0.028
Chair Sit-and-Reach (cm)	4.63 (1.01)	4.52 (1.07)	4.70 (0.97)	0.087
30 Seconds Arm Curl (reps)	2.21 (1.02)	2.13 (1.16)	2.26 (0.93)	0.024
2 Minutes Step (reps)	2.62 (1.03)	2.60 (1.03)	2.64 (0.97)	0.336
Back Scratch (cm)	3.66 (1.06)	3.53 (1.05)	3.74 (1.06)	0.009
8-Foot Up-and-Go (s)	2.76 (0.98)	2.86 (0.94)	2.70 (1.00)	0.06

### Multivariate Cox regression analysis of hypertension and HRPF

We utilized four Cox proportional hazards models to analyze each physical fitness indicator. Model 1: an unadjusted model; Model 2: adjusted for gender and age; Model 3: adjusted for exercise, smoking, and fall history; Model 4: adjusted for height, weight, BMI, hip-to-waist ratio (WHR), and heart rate. The result are presented in Table 4. In unadjusted Model 1, 30 s chair stand (β:−0.171, HR: 0.84, 95% CI: 0.74–0.97, *P* < 0.05), chair sit-and-reach (β:−0.122, HR: 0.88, 95% CI: 0.79–0.99, *P* < 0.05) and back scratch (β:−0.148, HR: 0.86, 95% CI: 0.77–0.97, *P* < 0.05) were identified as protective factors against hypertension. In adjusted Model 2, 30 s chair stand(β: −0.171 h༚0.84, 95% CI:0.74–0.97,*P* < 0.05), chair sit-and-reach(β༚−0.128, HR༚0.88, 95% CI:0.78–0.99, *P* < 0.05),back scratch(β:−0.149, HR༚0.86, 95% CI:0.77–0.97, *P* < 0.05) remained consistent with model 1, serving as significant protective effects. There were some changes in Model 3, 30 s chair stand (β:−0.155, HR: 0.86, 95% CI: 0.74–0.99, *P* < 0.05) and back scratch(β:−0.140, HR: 0.87, 95% CI: 0.77–0.97, *P* < 0.05) remained significant protective factors, however chair sit-and-reach test was no longer exhibited statistical significance in this model. In Model 4, none of the physical fitness indicators demonstrated statistical significance.

**Table 4:** Multivariate cox regression analysis on the hypertension and HRPF

**Column1**	*Model*	*β*	*HR(95%CI)*	*P*
One-Leg Balance	Model 1	0.058	1.06(0.94–1.19.94.19)	0.336
	Model 2	0.07	1.07(0.95–1.21.95.21)	0.249
	Model 3	0.099	1.10(0.98–1.25.98.25)	0.111
	Model 4	0.09	1.09(0.97–1.24.97.24	0.15
30 Seconds Chair Stand	Model 1	−0.171	0.84(0.74–0.97.74.97)	0.014
	Model 2	−0.167	0.85(0.74–0.97.74.97)	0.019
	Model 3	−0.155	0.86(0.74–0.99.74.99)	0.03
	Model 4	−0.127	0.88(0.77–1.01.77.01)	0.069
Chair Sit-and-Reach	Model 1	−0.122	0.88(0.79–0.99.79.99)	0.04
	Model 2	−0.128	0.88(0.78–0.99.78.99)	0.034
	Model 3	−0.119	0.89(0.79–1.00.79.00)	0.051
	Model 4	−0.091	0.91(0.81–1.03.81.03)	0.144
30 Seconds Arm Curl	Model 1	−0.119	0.89(0.77–1.03.77.03)	0.11
	Model 2	−0.114	0.89(0.77–1.04.77.04)	0.131
	Model 3	−0.078	0.93(0.79–1.08.79.08)	0.32
	Model 4	−0.065	0.94(0.80–1.10.80.10)	0.419
2 Minutes Step	Model 1	−0.027	0.97(0.86–1.10.86.10)	0.675
	Model 2	−0.016	0.98(0.87–1.12.87.12)	0.805
	Model 3	−0.01	0.99(0.87–1.12.87.12)	0.879
	Model 4	−0.008	1.01(0.89–1.15.89.15)	0.897
Back Scratch	Model 1	−0.148	0.86(0.77–0.97.77.97)	0.012
	Model 2	−0.149	0.86(0.77–0.97.77.97)	0.012
	Model 3	−0.14	0.87(0.77–0.97.77.97)	0.019
	Model 4	−0.089	0.91(0.81–1.04.81.04)	0.159
8-Foot Up-and-Go	Model 1	0.109	1.12(0.99–1.26.99.26)	0.08
	Model 2	0.106	1.11(0.98–1.26.98.26)	0.091
	Model 3	0.095	1.10(0.97–1.24.97.24)	0.131
	Model 4	0.075	1.08(0.95–1.23.95.23)	0.247

Model 1 unadjusted

Model 2 Adjusted for Gender and Age

Model 3 Adjusted for Exercise, Smoking and Fall Incidents

Model 4 Adjusted for Height, Weight, BMI, Hip Circumference, WHR and Heart Rate

## Discussion

This large-scale cohort study investigated the relationship between HRPF and incident hypertension among older adults. Multivariate Cox regression analysis indicated that better performance in 30 s chair stand, chair sit-and-reach and back scratch was associated with a reduced incidence of hypertension.

Notably, the association between lower-body flexibility, measured by the chair sit-and-reach test, and hypertension risk varied across adjustment models. It remained statistically significant in the unadjusted model and Model 2. However, the association became statistically non-significant after further adjustment in Model 3 and Model 4. This attenuation suggests that the observed association between lower body flexibility and hypertension may be partially mediated. In contrast to previous studies that often treat flexibility as a unified construct associated with reduced hypertension risk [25]. Our findings highlight the need to investigate the distinct roles and mechanisms of upper versus lower body flexibility separately.

Conversely, better upper-body flexibility, assessed via the back scratch test, was consistently associated with a lower risk of hypertension [19]. The robustness of the association for upper-body flexibility across all adjusted models highlights its potential as an independent protective factor. This result aligns with Japanese research that also identified an inverse relationship between good flexibility and hypertension incidence [20]. Another study involving 566 adults revealed that individuals over 40 years with poor flexibility exhibited systolic blood pressure values approximately 5 mmHg higher than those with good flexibility [21]. The mechanism underlying the relationship between flexibility and hypertension may involve in three intermediate parameters: arterial stiffness, arterial remodeling, and parasympathetic nervous activity dominance [22–25]. These factors induce alterations in vascular structure, arterial remodeling, or the release of vasodilatory metabolites, and such adaptive changes contribute to a reduction in vascular resistance, which may be associated with a lower incidence of hypertension [22–24, 26–28]. 

Our findings regarding lower body strength are consistent with existing literature. Numerous studies provide statistic evidence that higher relative muscle strength (RMS) is associated with a reduced risk of hypertension [15, 16]. This association remains significant in subgroups with diabetes, hyperlipidemia, and obesity, even after adjusting for covariates [15, 16]. Potential mechanisms that may explain the association between muscle strength and hypertension involve insulin resistance and skeletal muscle mass loss [29–31]. Insulin resistance may lead to sympathetic nervous system excitation, decreased renal vasoconstriction, and increased renal tubular sodium reabsorption, which are pathways that could potentially lead to elevated blood pressure [30, 32–34]. While loss of muscle mass may increase vascular damage and arterial stiffness, which may in turn elevate vascular resistance and be associated with a higher hypertension risk [31, 35–39]. 

Despite the observational nature of this study, our findings indicate that older adults engage in exercises aimed at enhancing body flexibility and lower body muscle strength could be explored as a potential strategy for hypertension prevention.

Previous research has emphasized the role of physical exercise in reducing chronic disease incidence and premature mortality among older adults [40, 41]. However, HRPF may represent a more significant factor than general physical activity. Compared with mere physical activity, HRPF is more robust risk factor cardiovascular disease. For instance, while increased physical activity is associated with an approximately 25% reduction in cardiovascular risk, improved physical fitness corresponds to about a 60% risk reduction [42]. Therefore, training programs targeting flexibility and muscular strength should be incorporated into both primary and secondary prevention strategies to enhance the effectiveness of hypertension prevention among the population.

For older adults, emphasizing physical exercise to enhance these two fitness dimensions is crucial for hypertension prevention. For muscle strength enhancement, resistance training and aerobic exercise are recommended [43, 44]. In terms of intensity, it is recommend that every week engaging in at least 150 min of moderate-intensity aerobic exercise or at least 75 min of vigorous-intensity aerobic exercise, or an combination of moderate-to-vigorous exercise at least 10 min every time [45]. To improve body flexibility, effective interventions include six-week static stretching programs, which involves performing bilateral stretches five times per week, with each session lasting 30 min and short-term Pilates training (5 or 10 weeks) or long-term Pilates training (6 months) and yoga are effectively [46–48]. Tai Chi and Square Stepping Exercise are particularly beneficial as they simultaneously enhance both flexibility and muscle strength [49]. – [50] Additionally, combining exercise with proper nutritional support has been shown better improvements [51]. 

Our findings have several important implications for public health initiatives and policy-making aimed at hypertension prevention in aging populations. Community-based exercise interventions should be integrated into public health strategies for older adults. Thus, we recommend that local health departments and community centers develop structured programs that simultaneously target both flexibility and muscle strength enhancement.From a policy perspective, our results support the inclusion of HRPF assessment in routine health screenings for older adults. Healthcare systems could consider incorporating simple fitness tests (such as the 30-second chair stand and back scratch tests) into annual health examinations for older adults.

## Conclusion

This large-scale cohort study demonstrates distinct relationships between specific dimensions of HRPF and hypertension in older adults. We observed that better lower muscle strength and up body flexibility were associated with a lower risk of hypertension. To potentially reduce the hypertension incidence and release the associated burden on the healthcare system, it may be more beneficial to focus on exercise training tailored to comprehensive HRPF indicators than to engage in non-specific physical activity.

## Limitation

This study has several limitations. First, as a cohort study, we encountered a high loss-to-follow-up rate of 26.8%. Among those lost, a considerable proportion likely died due to underlying health conditions, which may have excluded potential hypertension cases and introduced selection bias into the results. Second, some elderly participants had limited understanding of hypertension, possibly leading to inaccuracies in self-reporting and thus influencing the validity of the findings.

## Data Availability

The datasets generated and analysed during the current study are available from the corresponding author on reasonable request.

## Abbreviations

BP: Blood Pressure
CVD: Cardiovascular Disease
DBP: Diastolic Blood Pressure
HC: Hip Circumference
HRPF: Health-Related Physical Fitness
SBP: Systolic Blood Pressure
WC: Waist Circumference
WHR: Waist-to-Hip Ratio
YLL: Years of Life Lost
